# Seen on screen, missed in clinic: educational pearls from *House M.D.* – a reflective educational article on fictional cases in dermatology

**DOI:** 10.1093/skinhd/vzag035

**Published:** 2026-04-21

**Authors:** Mohammed Shanshal

**Affiliations:** Department of Dermatology, Imperial College Healthcare NHS Trust, London, UK

## Abstract

Dermatological clues are frequently overlooked in complex clinical presentations, both in real life and fictional portrayals. This article explores how seven dramatic cases from the television show *House M.D.* illustrate missed or delayed diagnoses where the skin held the key. Through structured analysis of each episode, this case-based educational review highlights how common dermatological signs, when recognized, could have expedited diagnosis. The review emphasizes the importance of skin assessment, especially in patients with systemic symptoms, skin of colour or subtle findings. By aligning entertainment with education, this review illustrates how fictional media can sharpen diagnostic reasoning and visual recognition, and suggests potential, unevaluated applications in dermatology teaching and journal club discussion.

## Teaser

This review examines seven dermatologic cases from the television show *House M.D.* where visible cutaneous signs were overlooked, leading to delayed or altered diagnoses. By contrasting on-screen portrayals with current evidence, we distil practical educational pearls on conditions such as erythropoietic protoporphyria, toxic epidermal necrolysis and melanoma in skin of colour. The article highlights how fictional media can serve as an engaging tool for teaching diagnostic vigilance and full-skin examination in clinical practice.

Dermatology is often under-represented or misrepresented in fictional media, yet televised medical dramas frequently feature skin findings that mirror real-life conditions. One prominent example is *House M.D.* (television series, 2004–12), a long-running series renowned for complex diagnoses and plotlines where subtle cutaneous clues play a decisive role.^1^ These portrayals, while dramatized, offer a unique opportunity for clinical reflection and diagnostic education.

In this case-based educational review, we explore seven dermatological diagnoses depicted in fictional scenarios, each aligned with real clinical entities. By analysing how these cases unfold (what was seen, what was missed and what could have been done differently) we highlight valuable diagnostic insights and teaching points relevant to everyday dermatological practice.

This article integrates visual recognition, diagnostic reasoning and interdisciplinary context, emphasizing how skin signs can serve as early or even life-saving clues. While drawn from scripted content, each case is anchored in scientific evidence and structured to reinforce practical clinical pearls. Our goal is to use fiction as a platform for real-world learning, reminding clinicians that the key to diagnosis is often visible but not always recognized.

## Methods

### Episode selection

All eight seasons of *House M.D.* were surveyed using a combination of the author’s prior viewing, targeted rewatching via streaming platforms, and publicly available episode guides and synopses to identify episodes with explicit dermatological content. Episodes were considered for inclusion if they (i) featured a recognizable cutaneous sign that was visible on screen or clearly described in the diagnostic narrative and (ii) linked that sign to a diagnosable dermatological or systemic condition in which the skin clue was missed, delayed or under-recognized. When multiple episodes illustrated similar concepts, a single exemplar with the clearest dermatological teaching point was retained, yielding seven vignettes as a purposive educational sample rather than an exhaustive catalogue of every skin appearance in the series.

### Case analysis and review process

For each selected episode, key scenes were rewatched to confirm the visibility, timing and clinical context of all depicted skin signs. The fictional trajectory was then compared with contemporary dermatology literature and relevant clinical practice guidelines for the corresponding condition, focusing on how accurately the cutaneous features and systemic manifestations were portrayed, where the on-screen work-up diverged from recommended practice, and which missed or delayed clues offered the strongest educational value.

### Realism commentary

For each vignette, the ‘Scientific insight’ section includes a brief realism commentary that considers three aspects of the portrayal: (i) diagnostic accuracy, (ii) how closely the presentation and management mirror real-world practice, and (iii) narrative plausibility. These comments draw out where the episode closely tracks clinical reality and where it takes dramatic licence, anchoring each case in current dermatology knowledge. These narrative comments are deliberately qualitative and are not a formal rating scale, and have not been psychometrically validated; they highlight where the episode aligns with or departs from current dermatology practice.

## Episode reviews and dermatological insights

### Case 1: ‘Finding Judas’ (season 3, episode 9): erythropoietic protoporphyria

#### Case presentation

A child is taken to surgery for severe abdominal pain of unknown cause. Under the bright surgical lights, he suddenly screams in agony, baffling the team. In past episodes, he had been dismissed as having heat intolerance or anxiety: he would cry in sunlight, despite having no visible skin changes. Only after this alarming intraoperative reaction does the team recognize the real trigger: a hidden photosensitivity. The diagnosis quite literally comes to light: erythropoietic protoporphyria (EPP).

#### Scientific insight

EPP is a rare autosomal recessive disorder caused primarily by mutations in *FECH*, resulting in deficient ferrochelatase activity and accumulation of protoporphyrin IX in erythrocytes and tissues.^2^ Clinically, EPP manifests with immediate burning pain, erythema and oedema upon exposure to sunlight, often without visible skin lesions, particularly early on.^3^ The episode accurately reflects this invisible yet severe phototoxicity, as the child’s acute distress occurs under high-intensity surgical lights without any apparent rash. While the episode illustrates phototoxicity vividly, the trigger (operating room lighting) is a dramatic embellishment. Exacerbations are overwhelmingly due to natural sunlight; case reports describe precautionary intraoperative light-filtering measures in cases of known EPP rather than actual injury in undiagnosed patients.^4–7^ The portrayal helps emphasize that phototoxicity in EPP is a neuropathic pain phenomenon, frequently misunderstood or misdiagnosed as anxiety or a behavioural disturbance.

#### Diagnosis and management

Diagnosis involves measuring fractionated erythrocyte protoporphyrin levels (metal-free vs. zinc-protoporphyrin), followed by genetic testing for *FECH* mutations when elevated.^8^ First-line management includes lifelong visible light photoprotection, using sun-protective clothing, physical blockers such as zinc oxide and minimizing exposure to blue–violet (≈400–410 nm) light, including artificial indoor sources; tinted window films and iron oxide–containing sunscreens can reduce transmission.^9^

Afamelanotide, a melanocortin-1 receptor agonist, has emerged as a disease-modifying therapy, improving quality of life and extending pain-free light exposure time in eligible patients.^10,11^ While β-carotene may reduce photosensitivity by increasing melanin density, evidence is inconsistent and its use is not routinely recommended.^9^ Vitamin D levels should be monitored and supplemented due to chronic light avoidance. Although the episode accurately portrays acute phototoxic distress, it misses an opportunity to model preventive counselling and long-term management strategies crucial to real-world care.

#### Educational pearls

- Pain without a rash after sun exposure? Think beyond sunburn: EPP causes intense burning or stinging minutes after light exposure, often without visible changes.^12^
- In children, EPP is often missed: early signs may be vague, such as crying in sunlight, avoiding outdoor play or hand swelling after sun exposure.
- Standard ultraviolet (UV) sunscreens do not work: EPP is triggered by visible light (especially blue–violet, ≈400–410 nm). Patients need iron oxide-tinted sunscreens, physical blockers, protective clothing and tinted window films.^9^
- Plasma porphyrins can be normal: diagnosis requires fractionated erythrocyte protoporphyrin testing (metal-free vs. zinc-protoporphyrin).^8^
- Clue in plain sight: recurrent summer symptoms without a rash should trigger a porphyria workup; painful photosensitivity with initially healthy-appearing skin suggests EPP.

### Case 2: ‘Locked In’ (season 5, episode 19): leptospirosis

#### Case presentation

A man is found locked in: conscious but unable to move. Clues emerge: jaundice, a rat-infested basement where he had been staying and a small periungual cut providing a portal of entry. The clinching moment in the episode is when a clinician develops a wrist rash after contact with the patient’s urine, prompting suspicion of a zoonosis acquired from urine-contaminated environments; the final diagnosis is leptospirosis, a stealthy zoonosis that nearly went unnoticed.

#### Scientific insight

Leptospirosis is a zoonosis acquired via contact with water or soil contaminated by infected animal urine, entering through skin cuts/abrasions or mucous membranes; macerated skin increases risk.^13^ Typical presentation includes abrupt fever, headache, severe calf-predominant myalgia and conjunctival suffusion (nonpurulent redness).^14^ Cutaneous manifestations are uncommon and short-lived, usually transient maculopapular or urticarial eruptions, sometimes with petechiae/purpura, or rarely erythema multiforme-like or erythema nodosum lesions.^15,16^ ‘Weil disease’ denotes the severe form with jaundice, acute kidney injury, haemorrhage (including pulmonary haemorrhage) and multi­organ involvement.^14^ In the episode, the wrist rash in a clinician after urine contact serves the plot; in practice, transmission to healthcare workers is rare, and leptospirosis has an incubation period typically of 7–12 days (range 2–20 days). An immediate local rash at the exposure site would be atypical.^17^

#### Diagnosis and management

Diagnosis requires a high index of suspicion with careful exposure history. Confirmation is by serology [IgM enzyme-linked immunosorbent assay (ELISA) or microscopic agglutination test as reference standard] or polymerase chain reaction (PCR) of blood in the first week, and later urine or cerebrospinal fluid in neurological cases.^18,19^ Treatment depends on severity: oral doxycycline for mild disease, intravenous (IV) penicillin G or ceftriaxone for severe cases, and amoxicillin as a safe option in pregnancy; azithromycin may be used when tetracyclines are contraindicated.^20,21^

#### Educational pearls

- Think leptospirosis after exposure to contaminated water or urine-contaminated environments (floodwater, lakes/rivers, sewers, waterlogged basements) with fever, calf-predominant myalgia and conjunctival suffusion.^14^
- Rats leave more than droppings. Leptospires are shed in rodent urine, and also by other mammals (e.g. dogs and livestock). Urban slums, farms and waterlogged basements are key exposure risks.^13^
- Red eyes without discharge? Conjunctival suffusion is a classic early clue, though not universal.
- A great imitator. Leptospirosis can mimic sepsis, hepatitis, aseptic meningitis or acute respiratory distress syndrome, especially in tropical or postflood settings.
- Not just a tropical disease. Cases occur in urban areas: sewer workers, campers, open-water athletes and people exposed to infected dogs are at risk.
- Start empirical antibiotics promptly when clinical suspicion is high; do not delay for labs – timely antibiotics can prevent progression to Weil disease.^21^

### Case 3: ‘A Pox on Our House’ (season 7, episode 7): rickettsialpox

#### Case presentation

A teenage diver developed fever and a papulovesicular rash after cutting her hand on glass fragments from an old jar recovered from a sunken ship that had carried enslaved peoples that had lain underwater for centuries, prompting hospital lockdown over feared smallpox. As part of containment, the family received smallpox vaccination; her father subsequently developed a widespread pustular eruption and died, escalating concern for variola. On post mortem examination, House identified a necrotic eschar, the overlooked dermatological clue that redirected the diagnosis to rickettsialpox, allowing the daughter to recover with doxycycline.

#### Scientific insight

Rickettsialpox, caused by *Rickettsia akari* and transmitted by the mouse mite (*Liponyssoides sanguineus*) from house mice, typically presents with fever, an inoculation eschar and a disseminated ­papulovesicular rash.^22^ The eschar, a sharply demarcated black crust with an erythematous halo, may be mistaken for an insect bite or minor bacterial infection, delaying recognition. The generalized rash can mimic viral exanthems such as varicella or even smallpox, but unlike variola it usually spares the palms and soles. Treatment with doxycycline is rapidly effective, with prompt clinical response.^23^

The episode takes dramatic licence: while variola virus can persist for months in dried scabs under cool, dark conditions,^24^ survival for centuries in submerged jars is implausible. Similarly, the father’s fulminant pustular decline immediately after vaccination is unrealistic: serious vaccinia complications such as generalized or progressive vaccinia arise days to weeks postvaccination,^25,26^ not within hours. These inaccuracies underscore the need to distinguish dramatic story­telling from dermatological fact, while highlighting the diagnostic importance of recognizing eschars in febrile rash syndromes.

#### Diagnosis and management

The diagnosis of rickettsialpox is primarily clinical, characterized by fever, papulovesicular rash and a preceding inoculation eschar. Confirmation is obtained retrospectively by demonstrating a fourfold rise in *R. akari* IgG antibody titres using indirect immunofluorescence assay, which remains the reference standard; PCR from eschar or blood can also provide supportive evidence.^27,28^ The main differential diagnosis is varicella. In contrast to the pleomorphic lesions of chickenpox that appear in various stages of evolution, the rash in rickettsialpox is typically monomorphic and ­papulovesicular, and it is almost always preceded by an eschar. A history of rodent infestation or mite exposure in an urban setting also favours rickettsialpox over chickenpox.^29,30^ Doxycycline is the treatment of choice, typically given for 5–7 days, with rapid clinical improvement expected; macrolides may be considered when tetracyclines are contraindicated.^27^

#### Educational pearls

- Look for the eschar. In febrile rash illness with possible arthropod exposure, a black inoculation eschar is a key but often-missed diagnostic clue for rickettsialpox and other eschar-­associated rickettsioses.^27^
- Urban ≠ safe. *Rickettsia akari* is transmitted by house-mouse mites in rodent-infested buildings; vector-borne illness can thrive in city apartments.^22^
- Chickenpox is the main mimic. Unlike varicella’s pleomorphic ‘crops’ of evolving vesicles, rickettsialpox produces a monomorphic papulovesicular rash, almost always preceded by an eschar.
- Eschar + rash = rethink ‘cellulitis’. A necrotic eschar mistaken for an insect bite, followed by a vesicular rash, should shift suspicion from bacterial cellulitis to rickettsialpox.
- Doxycycline cures fast. Doxycycline for 5–7 days is the treatment of choice, with rapid defervescence and recovery.^27^

### Case 4: ‘Unfaithful’ (season 5, Episode 15): Wiskott–Aldrich syndrome

#### Case presentation

A middle-aged priest is admitted after experiencing a sudden visual hallucination. During admission he develops toe necrosis with autoamputation, chest pain with numbness, acute monocular vision loss and a transient rash on the chest. Vascular imaging excludes embolic disease, but the discovery of *Pneumocystis jirovecii* infection prompts suspicion of an underlying immuno­deficiency. The overlooked clue is a history of lifelong bleeding: he had been ‘called a bleeder’ since childhood. Genetic testing confirms Wiskott–Aldrich syndrome (WAS).

#### Scientific insight

WAS is an X-linked inborn error of immunity caused by mutations in *WAS*, which encodes WASp, leading to defective actin cytoskeleton remodelling.^31^ The classic triad comprises recurrent infections, eczema and microthrombocytopenia (low platelet count with small platelets).^32,33^ Patients are predisposed to bleeding, autoimmunity, lymphoma and opportunistic infections such as *P. jirovecii*.^34–36^ In the episode, a transient truncal rash was shown and interpreted as a drug reaction; this is not a typical manifestation of WAS, where the cutaneous signs are classically eczema and petechiae or purpura from microthrombocytopenia.

The episode takes dramatic licence: toe autoamputation, hallucinations and acute monocular blindness are not manifestations of WAS. In practice, the overlooked clue, that of lifelong bleeding and being ‘called a bleeder’ since childhood, would have pointed much earlier to microthrombocytopenia. Diagnosis is confirmed by genetic testing for WAS, not the circuitous diagnostic pathway portrayed on screen.

These inaccuracies underscore the tension between drama and science, while highlighting a critical pearl: persistent bleeding and immunodeficiency, even in adults, should trigger suspicion for WAS or related primary immunodeficiencies.

#### Diagnosis and management

Diagnosis of WAS requires a ‘high index of suspicion’ in any patient with lifelong bleeding, petechiae, eczema and recurrent infections. The laboratory hallmark is thrombocytopenia with small platelets, which distinguishes WAS from other causes of thrombocytopenia.^37^ Although microthrombocytopenia is characteristic, rare patients with normal mean platelet volumes have been reported.^38,39^ Confirmation is achieved by demonstrating absent or reduced WASp expression on flow cytometry, and definitive diagnosis is by molecular testing of *WAS*.^40^

Supportive management includes bleeding precautions, infection prophylaxis, immunoglobulin replacement and skin care for eczema.^41^ Haematopoietic stem cell transplantation is the treatment of choice and should be performed early, offering long-term survival rates >80% in contemporary series.^42,43^ Gene therapy has shown durable benefit in clinical trials and represents an emerging curative option.^44,45^

#### Educational pearls

- Recurrent petechiae or easy bruising warrants investigation, especially with epistaxis or infections; assess platelet count, size and immune function.
- Eczema + infections + bleeding = think WAS: the classic triad of WAS should raise red flags, especially in men.^33^
- X-linked inheritance matters: a family history of male infant deaths, bleeding or infections supports the diagnosis.
- Small platelets with thrombocytopenia are the hallmark of WAS. Automated counters may misclassify microplatelets as debris and underestimate platelet counts, so confirmation on smear and mean platelet volume is essential.^32^
- Early diagnosis enables cure: haematopoietic stem cell transplant is curative; immunology referral is urgent.^42,43^

### Case 5: ‘Under My Skin’ (season 5, episode 23): toxic epidermal necrolysis

#### Case presentation

After empirical antibiotics for suspected infection, a young ballerina’s ‘simple drug allergy’ rapidly declared itself as sheet-like epidermal sloughing, ultimately involving ∼80% body surface area. She was transferred to the burn unit and managed with synthetic dressings. Dermatology was not consulted on screen. The culprit reaction was recognized as toxic epidermal necrolysis (TEN).

#### Scientific insight

TEN is a severe drug-induced mucocutaneous reaction defined by >30% body surface epidermal detachment with massive keratinocyte necrosis.^46,47^ Mucosal involvement is a usual feature,^48^ although it was not depicted in the episode. Patients typically report severe skin pain followed by rapid sheet-like detachment after a recent high-risk medication.^49^ The episode accurately conveys the terrifying speed and severity of TEN, escalating from a ‘simple drug allergy’ to widespread epidermal sloughing. However, it contains two significant deviations from clinical reality. Firstly, it omits the severe mucosal involvement (oral, ocular, genital) that is a hallmark of TEN in over 90% of cases. Secondly, and perhaps most critically from a systems-based practice perspective, the patient’s care is managed without any on-screen consultation from a dermatology service. In real-world practice, early involvement of dermatology is considered the standard of care and is crucial for accurate diagnosis, biopsy and coordinating complex multidisciplinary care. This omission in the show serves as a powerful ‘negative’ teaching point about the importance of interspecialty collaboration in managing dermatological emergencies.

#### Diagnosis and management

Early recognition is crucial: painful erythema with rapid epidermal detachment and mucosal ulceration are the cardinal clinical clues.^46,47^ Histology shows full-thickness epidermal necrosis with sparse dermal inflammation, which helps distinguish TEN from other blistering disorders.^50,51^ Severity and prognosis should be assessed using the SCORTEN (Severity-of-Illness Score for Toxic Epidermal Necrolysis) score, which is validated for predicting mortality.^52,53^

Management hinges on immediate withdrawal of the offending drug, which is the single most important intervention. Patients require specialist unit care [burn/intensive care unit (ICU)/­dermatology] for meticulous supportive therapy, including fluid and ­electrolyte balance, wound care, temperature control, nutritional ­support and infection prevention.^54–56^ The benefit of ­systemic immunomodulators (e.g. ciclosporin, etanercept, intravenous immunoglobulin and corticosteroids) remains debated, with no universal consensus; supportive care remains the cornerstone.^57,58^

#### Educational pearls

- Pain before peel is the warning sign: TEN often begins with skin pain/tenderness before any blistering; rapid sheet-like detachment follows.
- Mucosa tells the story early: ulceration of the mouth, eyes or genitals occurs in >90% of cases and may precede skin detachment.^48^
- Nikolsky positive? Do not wait: gentle pressure lifting the epidermis signals necrolysis (not specific, also in pemphigus and staphylococcal scalded skin syndrome), but in suspected TEN it means stop all culprit drugs immediately.
- Never wait for biopsy to act: histology confirms, but early drug withdrawal is the single most important intervention.
- TEN is a medical emergency: TEN requires ICU or burns unit-level care; supportive management saves lives. It is never ‘just a rash’.
- Dermatology matters: unlike the episode, real practice mandates early dermatology input for diagnosis, biopsy interpretation and care coordination.
- SCORTEN predicts mortality: calculate within 24 h to risk-stratify and guide level of care.^52,53^
- Eyes are hit early: get ophthalmology urgently. Ocular disease is common, and early specialist care reduces the risk of chronic damage (e.g. symblepharon and vision loss).^59^

### Case 6: ‘Moving the Chains’ (season 6, episode 13): acral melanoma

#### Case presentation

A 22-year-old Black collegiate football player is admitted after a violent outburst with amnesia. During admission he develops paroxysmal tachycardia, and acute renal dysfunction. Extensive imaging fails to identify a primary malignancy, but suspicion of a paraneoplastic process persists. Only after examining his extremities is a small, pigmented macule discovered between the toes on the plantar foot. The overlooked primary: acral melanoma, a diagnosis often delayed in patients with skin of colour.

#### Scientific insight

Acral lentiginous melanoma (ALM) is the most common melanoma subtype in patients with skin of colour, representing the majority of melanomas in Black and Asian populations, and disproportionately affecting Hispanic patients.^60,61^ It arises on palms, soles and nail units, sites often underexamined and underappreciated during routine checks.^62^ ALM is typically diagnosed at a later stage and greater Breslow thickness than nonacral melanoma, due to both anatomical concealment and low clinical suspicion in darker skin.^63–65^ Chronic mechanical stress and repetitive trauma at weight-bearing or sports-related pressure points (such as in football players) are increasingly recognized as preferential sites for ALM.^66^

In the episode, the diagnosis is made only after a workup for a paraneoplastic syndrome points towards a hidden malignancy. This narrative reflects a common real-world pitfall: acral lesions in patients with skin of colour are often overlooked until the disease is advanced. A critical omission in the fictional workup was the failure to consult dermatology and perform a basic dermatological examination with dermoscopy.

#### Diagnosis and management

Diagnosis is clinical and supported by dermoscopy, with patterns such as the parallel ridge pattern or irregular fibrillar lines raising strong suspicion.^67,68^ Confirmation is by histopathology with immunohistochemistry (e.g. S-100, SOX10, Melan-A and HMB-45).^69,70^ Early detection is critical, as acral melanomas are typically diagnosed at greater Breslow thickness than nonacral subtypes.^63,64^ Molecularly, ALM often lacks *BRAF* mutations but may harbour *KIT* alterations, which can influence systemic therapy in advanced disease.^71–73^ Management follows standard melanoma staging protocols: wide local excision, sentinel lymph node assessment where appropriate, and systemic therapy guided by stage and molecular profile.^74–76^

#### Educational pearls

- Melanoma does not always mean sun: ALM occurs on palms, soles and nail units and is not UV-driven.^77^
- Low incidence ≠ low risk: ALM is rarer overall but shows worse stage at diagnosis and survival than nonacral cutaneous melanoma, largely from diagnostic delay and thickness.^78^
- Think beyond moles: warts, bruises and tinea are common mislabels; a changing acral pigmented lesion warrants evaluation.
- Ridges = risk, furrows = fine: on soles, the parallel ridge pattern on dermoscopy is highly predictive of melanoma; the parallel furrow pattern is usually benign and typical of acral naevi.^79–81^
- Acral surfaces must be checked: palms/soles/nails are easily missed without a full skin exam, even in hospital.

### Case 7: ‘Ugly’ (season 4, episode 7): Lyme disease

#### Case presentation

A teenage boy with a large facial mass is admitted for corrective surgery but develops cardiac conduction abnormalities and later a major gastrointestinal bleed, prompting multiple competing differentials. Just before surgery, shaving his scalp reveals a concealed target-like rash at the hairline, redirecting the diagnosis to Lyme disease and sparing him an unnecessary operation.

#### Scientific insight

Lyme disease is a multisystem infection that may manifest with cardiac conduction defects, neurological changes or systemic features, but the hallmark clue is erythema migrans (EM).^82,83^ When EM occurs on the scalp or other hair-bearing sites, it can remain hidden and delay recognition.^84^ More broadly, the scalp is a notoriously underexamined area that may conceal serious infections, inflammatory disorders, or even cutaneous malignancies.^85,86^ The episode compresses disease stages: EM is classically a marker of early localized Lyme disease, whereas atrioventricular block reflects early disseminated disease. Disseminated Lyme disease can occur with or without EM, and when rash is present at this stage it may be single or multiple.^87,88^ By telescoping early and disseminated features into the same window, the show heightens drama but still conveys the critical dermatological message: a hidden scalp rash can be the decisive diagnostic clue.

#### Diagnosis and management

Lyme disease is diagnosed on clinical grounds when EM is present, as this pathognomonic rash alone is sufficient to establish the diagnosis and begin treatment.^89,90^ In patients without EM or with later manifestations, confirmation relies on two-tier serological testing, consisting of an initial enzyme immunoassay (EIA)/ELISA followed by a confirmatory immunoblot or a second EIA (modified two-tier algorithm).^91–93^

Treatment depends on stage. Early localized disease is managed with oral doxycycline, amoxicillin or cefuroxime for 10–21 days. Disseminated or late disease requires longer courses, and in systemic involvement, IV ceftriaxone for 2–4 weeks is typically recommended.^89,93,94^ Prognosis is generally excellent when antibiotics are initiated promptly.^95^

#### Educational pearls

- Look for EM: the hallmark rash in most patients; but a minority may lack it, especially in disseminated disease.^90^
- Ticks are stealthy: most patients never recall a bite, and scalp sites are easily missed beneath hair.^90^
- Serology confirms, but history diagnoses: two-tier testing (ELISA + Western blot) is standard but consider clinical suspicion in endemic regions, even when tests lag behind.^89^
- Skin signs often precede systemic symptoms: dermatologists can spot early Lyme. A full skin exam, including scalp, may have revealed the diagnosis earlier; do not skip it.

## Discussion

### Media as a pedagogic tool in dermatology

While *House M.D.* is a fictional drama, this article uses it as a lens to explore how dermatological signs are portrayed, overlooked or correctly leveraged in complex diagnostic narratives. Yet fiction can mislead. When consumed passively, it risks promoting clinical misinformation or oversimplified reasoning. Cultivating critical appraisal is therefore essential, transforming scripted drama into a tool for thoughtful reflection rather than a source of unchecked truths.^96,97^ When analysed critically, such portrayals can become effective teaching tools for reinforcing pattern recognition, clinical reasoning and interdisciplinary thinking in dermatology training.

### Across these seven cases, a common thread emerged

Cutaneous clues were present but often under-recognized, resulting in delayed or missed diagnoses. These missed clues reflect real-world diagnostic pitfalls. In many cases, systemic symptoms distracted from skin findings, especially when the signs were subtle or atypical. These patterns highlight the critical role of dermatology in multidisciplinary care and underscore how fiction can serve as a reflective educational platform when analysed through a clinical lens. The acral melanoma case, for example, underscores the diagnostic challenges posed by subtle lesions in richly pigmented skin, where classic erythema or pigmentation change may be less readily visible. This pattern is mirrored in the literature: cutaneous signs often serve as early systemic clues, yet public awareness is limited. Fictional medical dramas, which are widely consumed, can both inform and mislead, underscoring the need for critical appraisal in education.^98–101^

### Educational impact on trainees

For trainees, fictional cases offer a low-cost, high-engagement complement to traditional dermatology teaching, particularly for practising pattern recognition, diagnostic reasoning and communication around uncertainty. In this article the seven vignettes are used with three practical learning objectives: (i) to heighten awareness of cutaneous clues in multisystem disease, (ii) to model critical appraisal of on-screen dermatological portrayals against contemporary evidence, and (iii) to provide structured material for small-group discussion of visual diagnosis and diagnostic reasoning. These aims align with case-based learning and guided reflection mapped broadly to the understand, apply and analyse levels of Bloom’s revised taxonomy. Table 1 therefore functions as a one-page teaching plan, summarizing for each episode the key skin clue, the diagnostic pitfall, a focused teaching pearl and a simple activity that can be used in journal-club or flipped-classroom formats.^102^

**Table 1:** *House M.D.* dermatology vignettes as teaching tools

Episode and diagnosis	Key cutaneous clue	What was missed	Educational pearls	Suggested activity (journal club/flipped class)
S3E9 ‘Finding Judas’: EPP	Immediate, severe burning pain on light exposure with little or no visible rash	Recurrent photosensitivity episodes were dismissed as behavioural/anxiety, without targeted history or porphyria work-up	Painful photosensitivity without rash, especially in a child who ‘hates the sun’, should prompt EPP testing; standard UV sunscreens are not protective	Show a brief written or video vignette of the preoperative light reaction and ask learners to list differential diagnoses and appropriate investigations for EPP
S5E19 ‘Locked In’: leptospirosis	New eruption at the wrist where a staff member was splashed with the patient’s urine, signalling infectious urine exposure	Early work-up focused on neurological/toxic causes and did not connect the rodent-infested basement and urine exposure to a possible zoonosis; leptospirosis was only considered after the contact eruption	A careful exposure history, combined with new lesions at points of contact, can unmask zoonotic infections such as leptospirosis, even in nontropical settings	Discuss how rodent-infested housing and a new lesion at a urine-splash site should prompt suspicion of leptospirosis
S7E7 ‘A Pox on Our House’: rickettsialpox	Inoculation eschar with papulovesicular rash mimicking chickenpox/smallpox	The eschar was overlooked and the eruption framed as possible smallpox, escalating to unnecessary high-level isolation	Eschar plus fever and vesicular rash, especially in settings with rodent infestation, should prompt suspicion for rickettsialpox and related rickettsioses	Present a minivignette of a patient with fever and an inoculation eschar; ask learners to list the differential diagnoses and decide whether they would start empiric doxycycline
S5E15 ‘Unfaithful’: WAS	Eczematous rash on the chest in a man with a lifelong history of easy bruising and bleeding	Eczema with lifelong bleeding was not recognized as a clue to inherited immunodeficiency; WAS entered the differential only after an opportunistic infection prompted an HIV-focused work-up	Eczema, infections and bleeding in a male patient should prompt early consideration of WAS and related inborn errors, even in adults	Give the lifetime bleeding history, current opportunistic infection and eczematous rash plus a full blood count (highlighting low platelets); ask trainees which primary immunodeficiencies they would consider and what tests they would order
S5E23 ‘Under My Skin’: TEN	Rapidly progressive, exquisitely painful sheet-like epidermal detachment after new medication	The eruption was initially treated as a simple drug reaction; recognition of TEN and escalation of care were delayed and no dermatology input shown	‘Pain before peel’ and mucosal involvement are red flags for TEN; early culprit drug withdrawal and urgent dermatology/ICU referral are critical	Using still images or a written vignette, have learners calculate SCORTEN, list probable culprit drugs and outline the first 24 h of real-world TEN management
S6E13 ‘Moving the Chains’: acral melanoma	Small, pigmented macule between the toes on the plantar foot of a patient with skin of colour	A full skin examination was deferred; the acral lesion was discovered only after a paraneoplastic search suggested an occult malignancy	Acral melanoma is a common melanoma subtype in patients with skin of colour and often presents late because soles and nail units are underexamined	Provide clinical and dermoscopic images of multiple acral lesions and ask learners to decide which need biopsy
S4E7 ‘Ugly’: Lyme disease	Erythema migrans-like targetoid lesion hidden beneath scalp hair	The scalp was not examined until surgical preparation, so the diagnostic rash was recognized only after invasive planning	Hair-bearing areas such as the scalp can conceal key rashes; a deliberate, head-to-toe skin examination is essential in multisystem presentations	Present a vignette of a young patient from a Lyme-endemic area with AV block and facial palsy where ‘no rash’ is documented; ask whether this rules out Lyme disease and which hidden sites should be specifically re-examined

AV block, atrioventricular block; E, episode; EPP, erythropoietic protoporphyria; ICU, intensive care unit; S, series; SCORTEN, Severity-of-Illness Score for Toxic Epidermal Necrolysis; TEN, toxic epidermal necrolysis; UV, ultraviolet; WAS, Wiskott–Aldrich syndrome.

### Public perception and specialty awareness

Dermatology’s portrayal in popular culture shapes public expectations and can influence the perceived prestige of the field. When accurately depicted, as in cases of lupus, porphyria or Ehlers–Danlos syndrome, the educational ripple effect extends beyond clinicians to patients, enhancing understanding of chronic disease visibility, stigma and diagnostic complexity. Accurate depictions of, for example, acral melanoma, Lyme disease and TEN can help reduce diagnostic delay, destigmatize visible conditions, and foster empathy among nondermatologists and patients alike.

### Future directions for collaboration

There is untapped potential for collaboration between dermatologists and media creators. Involving dermatology experts in script development, storyline consultation or brief postepisode commentaries could significantly enhance medical accuracy without disrupting dramatic pacing. These partnerships would not only improve public health literacy, but also help elevate dermatology’s profile as a diagnostic cornerstone in complex medical cases. Involving dermatology experts in media production could mirror collaborations seen in infectious disease storylines, such as Centers for Disease Control and Prevention consultation on *Contagion*, to enhance accuracy without compromising entertainment value.

## Limitations

This work has several important limitations. It is a single-author, purposive selection of seven episodes from one television series and is not intended to be an exhaustive survey of dermatology in popular media. The episode selection, interpretation of cutaneous signs and ‘realism commentary’ are inherently subjective and may reflect the author’s clinical and educational biases. No formal evaluation data are available regarding the impact of using these vignettes in teaching, so the suggested activities should be viewed as illustrative rather than evidence-based recommendations.

## Conclusion

*House M.D.* offers a unique narrative space where dermatological signs, often overlooked in both fiction and practice, are central to unravelling diagnostic puzzles. This case-based educational review demonstrates how critically analysing fictional portrayals through a clinical lens can sharpen diagnostical vigilance, reinforce core dermatological principles and promote reflective learning. By aligning entertainment with education, such media can highlight diagnostic blind spots and strengthen the skin’s role as a vital diagnostic tool. Integrating fictional case analysis into dermatology education may offer a low-cost, high-engagement adjunct to traditional training, but at present this remains a proposed use rather than an evaluated teaching intervention. Formal educational studies would be required to determine whether using such vignettes measurably improves diagnostic reasoning or recognition of critical skin signs.

## References

[1] *House, M.D. [television series]*. Los Angeles: Fox Broadcasting Company, 2004–2012.

[2] Graham-Brown MP, Ilchyshyn A. Development of acute phototoxic reaction during surgery in a patient with erythropoietic protoporphyria. Clin Exp Dermatol 2013; 38:566–8. 23777502 10.1111/ced.12188

[3] Poli A, Schmitt C, Puy H et al Erythropoietic protoporphyrias: updates and advances. Trends Mol Med 2024; 30:863–74. 38890030 10.1016/j.molmed.2024.05.006

[4] Hanaki T, Noda T, Eguchi H et al Successful liver transplantation for liver failure with erythropoietic protoporphyria by covering the operating theater lights with polyimide film: a case report. Transplant Proc 2020; 52:625–9. 32029313 10.1016/j.transproceed.2019.12.004

[5] Pielaciński K, Kwasny M, Górski W et al Protection from phototoxic injury during laparoscopic surgery in patients with erythropoietic protoporphyria. Wideochir Inne Tech Maloinwazyjne 2022; 17:385–6. 35707342 10.5114/wiitm.2022.115003 PMC9186081

[6] Wahlin S, Srikanthan N, Hamre B et al Protection from phototoxic injury during surgery and endoscopy in erythropoietic protoporphyria. Liver Transpl 2008; 14:1340–6. 18756472 10.1002/lt.21527

[7] Shimazaki A, Hashimoto T, Kai M et al Surgical treatment for breast cancer in a patient with erythropoietic protoporphyria and photosensitivity: a case report. Surg Case Rep 2021; 7:1. 33400006 10.1186/s40792-020-01068-5 PMC7785757

[8] Minder AE, Kluijver LG, Barman-Aksözen J et al Erythropoietic protoporphyrias: pathogenesis, diagnosis and management. Liver Int 2025; 45:e16027. 39011756 10.1111/liv.16027 PMC11669082

[9] Dickey AK, Naik H, Keel SB et al Evidence-based consensus guidelines for the diagnosis and management of erythropoietic protoporphyria and X-linked protoporphyria. J Am Acad Dermatol 2023; 89:1227–37. 36041558 10.1016/j.jaad.2022.08.036 PMC9968824

[10] Langendonk JG, Balwani M, Anderson KE et al Afamelanotide for erythropoietic protoporphyria. N Engl J Med 2015; 373:48–59. 26132941 10.1056/NEJMoa1411481 PMC4780255

[11] Wensink D, Wagenmakers MA, Langendonk JG. Afamelanotide for prevention of phototoxicity in erythropoietic protoporphyria. Expert Rev Clin Pharmacol 2021; 14:151–60. 33507118 10.1080/17512433.2021.1879638

[12] Lecluse AL, Kuck-Koot VC, Van Weelden H et al Erythropoietic protoporphyria without skin symptoms-you do not always see what they feel. Eur J Pediatr 2008; 167:703–6. 17710435 10.1007/s00431-007-0557-1 PMC2292482

[13] Bharti AR, Nally JE, Ricaldi JN et al Leptospirosis: a zoonotic disease of global importance. Lancet Infect Dis 2003; 3:757–71. 14652202 10.1016/s1473-3099(03)00830-2

[14] Daher EF, Lima RS, Júnior GB et al Clinical presentation of leptospirosis: a retrospective study of 201 patients in a metropolitan city of Brazil. Braz J Infect Dis 2010; 14:3–10. 20428646

[15] Haake DA, Levett PN. Leptospirosis in humans. Curr Top Microbiol Immunol 2015; 387:65–97. 25388133 10.1007/978-3-662-45059-8_5 PMC4442676

[16] Davoodi L, Kazeminejad A, Jafarpour H et al Leptospirosis presented with erythema nodosum on four limbs: an unusual presenting. Oxf Med Case Rep 2020; 2020:omz130. 10.1093/omcr/omz130 PMC6996038 32038875

[17] Rajapakse S . Leptospirosis: clinical aspects. Clin Med 2022; 22:14–17. 10.7861/clinmed.2021-0784 PMC8813018 35078790

[18] Pinto GV, Senthilkumar K, Rai P et al Current methods for the diagnosis of leptospirosis: issues and challenges. J Microbiol Methods 2022; 195:106438. 35248601 10.1016/j.mimet.2022.106438

[19] Valente M, Bramugy J, Keddie SH et al Diagnosis of human leptospirosis: systematic review and meta-analysis of the diagnostic accuracy of the Leptospira microscopic agglutination test, PCR targeting Lfb1, and IgM ELISA to *Leptospira fainei* serovar Hurstbridge. BMC Infect Dis 2024; 24:168. 38326762 10.1186/s12879-023-08935-0 PMC10848445

[20] Ji Z, Jian M, Su X et al Efficacy and safety of antibiotics for treatment of leptospirosis: a systematic review and network meta-analysis. Syst Rev 2024; 13:108. 38627798 10.1186/s13643-024-02519-y PMC11020203

[21] Win TZ, Han SM, Edwards T et al Antibiotics for treatment of leptospirosis. Cochrane Database Syst Rev 2024; 2024:CD014960. 10.1002/14651858.CD014960.pub2 PMC10938876 38483092

[22] Eremeeva M, Muniz-Rodriguez K. Rickettsialpox – a rare but not extinct disease: a review of the literature and new directions. Russ J Infect Immun 2020; 10:477–85.

[23] Saini R, Pui JC, Burgin S. Rickettsialpox: report of three cases and a review. J Am Acad Dermatol 2004; 51:S137–42. 15577753 10.1016/j.jaad.2004.03.036

[24] McCollum AM, Li Y, Wilkins K et al Poxvirus viability and signatures in historical relics. Emerg Infect Dis 2014; 20:177–84. 24447382 10.3201/eid2002.131098 PMC3901489

[25] Casey CG, Iskander JK, Roper MH et al Adverse events associated with smallpox vaccination in the United States, January–October 2003. JAMA 2005; 294:2734–43. 16333009 10.1001/jama.294.21.2734

[26] Rotz LD, Dotson DA, Damon IK et al Vaccinia (smallpox) vaccine; recommendations of the Advisory Committee Immunization Practices (ACIP), 2001. MMWR Recomm Rep 2001; 50:1–25. 15580803

[27] Biggs HM . Diagnosis and management of tickborne rickettsial diseases: Rocky Mountain spotted fever and other spotted fever group rickettsioses, ehrlichioses, and ­anaplasmosis – United States. MMWR Recomm Rep 2016; 65:1–44. 10.15585/mmwr.rr6502a1 27172113

[28] Parola P, Paddock CD, Socolovschi C et al Update on tick-borne rickettsioses around the world: a geographic approach. Clin Microbiol Rev 2013; 26:657–702. 24092850 10.1128/CMR.00032-13 PMC3811236

[29] Rao M . Rickettsialpox or chickenpox? IDCases 2021; 25:e01138. 34094862 10.1016/j.idcr.2021.e01138 PMC8164020

[30] Diaz JH . Bitten. Wilderness Environ Med 2023; 34:589–92. 37598019 10.1016/j.wem.2023.06.009

[31] Bosticardo M, Marangoni F, Aiuti A et al Recent advances in understanding the pathophysiology of Wiskott–Aldrich syndrome. Blood 2009; 113:6288–95. 19351959 10.1182/blood-2008-12-115253

[32] Notarangelo LD, Miao CH, Ochs HD. Wiskott–Aldrich syndrome. Curr Opin Hematol 2008; 15:30–6. 18043243 10.1097/MOH.0b013e3282f30448

[33] Ochs HD, Thrasher AJ. The Wiskott–Aldrich syndrome. J Allergy Clin Immunol 2006; 117:725–38. 16630926 10.1016/j.jaci.2006.02.005

[34] Dupuis-Girod S, Medioni J, Haddad E et al Autoimmunity in Wiskott–Aldrich syndrome: risk factors, clinical features, and outcome in a single-center cohort of 55 patients. Pediatrics 2003; 111:e622–7. 12728121 10.1542/peds.111.5.e622

[35] Keszei M, Kritikou JS, Sandfort D et al Wiskott–Aldrich syndrome gene mutations modulate cancer susceptibility in the p53 ± murine model. Oncoimmunology 2018; 7:e1468954. 30393584 10.1080/2162402X.2018.1468954 PMC6209425

[36] Candotti F . Clinical manifestations and pathophysiological mechanisms of the Wiskott–Aldrich syndrome. J Clin Immunol 2018; 38:13–27. 29086100 10.1007/s10875-017-0453-z

[37] Cavannaugh C, Ochs HD, Buchbinder D. Diagnosis and clinical management of Wiskott–Aldrich syndrome: current and emerging techniques. Expert Rev Clin Immunol 2022; 18:609–23. 35533396 10.1080/1744666X.2022.2074400

[38] Mawalla WF, Iddy H, Kindole CA et al Wiskott–Aldrich syndrome with normal platelet volume in a low-income setting: a case report. Ther Adv Rare Dis 2021; 2:26330040211009905. 37181115 10.1177/26330040211009905 PMC10032462

[39] Patel PD, Samanich JM, Mitchell WB et al A unique presentation of Wiskott–Aldrich syndrome in relation to platelet size. Pediatr Blood Cancer 2011; 56:1127–9. 21488158 10.1002/pbc.22920

[40] Ochs HD, Filipovich AH, Veys P et al Wiskott–Aldrich syndrome: diagnosis, clinical and laboratory manifestations, and treatment. Biol Blood Marrow Transplant 2009; 15:84–90. 19147084 10.1016/j.bbmt.2008.10.007

[41] Rivers E, Worth A, Thrasher AJ et al How I manage patients with Wiskott Aldrich syndrome. Br J Haematol 2019; 185:647–55. 30864154 10.1111/bjh.15831 PMC7612067

[42] Albert MH, Slatter MA, Gennery AR et al Hematopoietic stem cell transplantation for Wiskott–Aldrich syndrome: an EBMT Inborn Errors Working Party analysis. Blood 2022; 139:2066–79. 35100336 10.1182/blood.2021014687

[43] Burroughs LM, Petrovic A, Brazauskas R et al Excellent outcomes following hematopoietic cell transplantation for Wiskott–Aldrich syndrome: a PIDTC report. Blood 2020; 135:2094–105. 32268350 10.1182/blood.2019002939 PMC7273831

[44] Boztug K, Schmidt M, Schwarzer A et al Stem-cell gene therapy for the Wiskott–Aldrich syndrome. N Engl J Med 2010; 363:1918–27. 21067383 10.1056/NEJMoa1003548 PMC3064520

[45] Abina SH, Gaspar HB, Blondeau J et al Outcomes following gene therapy in patients with severe Wiskott–Aldrich syndrome. JAMA 2015; 313:1550–63. 25898053 10.1001/jama.2015.3253 PMC4942841

[46] Pereira FA, Mudgil AV, Rosmarin DM. Toxic epidermal necrolysis. J Am Acad Dermatol 2007; 56:181–200. 17224365 10.1016/j.jaad.2006.04.048

[47] Downey A, Jackson C, Harun N et al Toxic epidermal necrolysis: review of pathogenesis and management. J Am Acad Dermatol 2012; 66:995–1003. 22169256 10.1016/j.jaad.2011.09.029

[48] Lissia M, Mulas P, Bulla A et al Toxic epidermal necrolysis (Lyell’s disease). Burns 2010; 36:152–63. 19766401 10.1016/j.burns.2009.06.213

[49] Mockenhaupt M, Viboud C, Dunant A et al Stevens–Johnson syndrome and toxic epidermal necrolysis: assessment of medication risks with emphasis on recently marketed drugs. The EuroSCAR-Study. J Invest Dermatol 2008; 128:35–44. 17805350 10.1038/sj.jid.5701033

[50] Valeyrie-Allanore L, Bastuji-Garin S, Guégan S et al Prognostic value of histologic features of toxic epidermal necrolysis. J Am Acad Dermatol 2013; 68:e29–35. 22088428 10.1016/j.jaad.2011.10.007

[51] Quinn AM, Brown K, Bonish BK et al Uncovering histologic criteria with prognostic significance in toxic epidermal necrolysis. Arch Dermatol 2005; 141:683–7. 15967913 10.1001/archderm.141.6.683

[52] Fouchard N, Bertocchi M, Roujeau JC et al SCORTEN: a severity-of-illness score for toxic epidermal necrolysis. J Invest Dermatol 2000; 115:149–53. 10951229 10.1046/j.1523-1747.2000.00061.x

[53] Torres-Navarro I, Briz-Redón Á, Botella-Estrada R. Accuracy of SCORTEN to predict the prognosis of Stevens–Johnson syndrome/toxic epidermal necrolysis: a systematic review and meta-analysis. J Eur Acad Dermatol Venereol 2020; 34:2066–77. 31912590 10.1111/jdv.16137

[54] Brüggen MC, Le ST, Walsh S et al Supportive care in the acute phase of Stevens–Johnson syndrome and toxic epidermal necrolysis: an international, multidisciplinary Delphi-based consensus. Br J Dermatol 2021; 185:616–26. 33657677 10.1111/bjd.19893

[55] Fernando SL . The management of toxic epidermal necrolysis. Australas J Dermatol 2012; 53:165–71. 22881464 10.1111/j.1440-0960.2011.00862.x

[56] Shah H, Parisi R, Mukherjee E et al Update on Stevens–Johnson syndrome and toxic epidermal necrolysis: diagnosis and management. Am J Clin Dermatol 2024; 25:891–908. 39278968 10.1007/s40257-024-00889-6 PMC11511757

[57] Tsai TY, Huang IH, Chao YC et al Treating toxic epidermal necrolysis with systemic immunomodulating therapies: a systematic review and network meta-analysis. J Am Acad Dermatol 2021; 84:390–7. 32898587 10.1016/j.jaad.2020.08.122

[58] Kridin K, Brüggen MC, Chua SL et al Assessment of treatment approaches and outcomes in Stevens–Johnson syndrome and toxic epidermal necrolysis: insights from a pan-­European multicenter study. JAMA Dermatol 2021; 157:1182–90. 34431984 10.1001/jamadermatol.2021.3154 PMC8387938

[59] Tóth G, Lukács A, Schirra F et al Ophthalmic aspects of Stevens–Johnson syndrome and toxic epidermal necrolysis: a narrative review. Ophthalmol Ther 2023; 12:1795–811. 37140876 10.1007/s40123-023-00725-w PMC10157599

[60] Nadelmann ER, Singh AK, Abbruzzese M et al Acral melanoma in skin of color: current insights and future directions: a narrative review. Cancers (Basel) 2025; 17:468. 40805297 10.3390/cancers17152458 PMC12345907

[61] Marchetti MA, Chung E, Halpern AC. Screening for acral lentiginous melanoma in dark-skinned individuals. JAMA Dermatol 2015; 151:1055–6. 26083954 10.1001/jamadermatol.2015.1347

[62] Skudalski L, McMullan P, Grant-Kels JM. Melanoma in patients with skin of color. Clin Dermatol 2025; 43:48–55. 39900308 10.1016/j.clindermatol.2025.01.014

[63] Grant S, Revan D, Tang L et al Disparities in acral lentiginous melanoma: racial factors beyond delayed diagnosis. J Am Acad Dermatol 2025; 93:1018–26. 40562077 10.1016/j.jaad.2025.06.048

[64] Cassalia F, Danese A, Cocchi E et al Misdiagnosis and clinical insights into acral amelanotic melanoma – a systematic review. J Pers Med 2024; 14:518. 38793100 10.3390/jpm14050518 PMC11121852

[65] Bello DM, Chou JF, Panageas KS et al Prognosis of acral melanoma: a series of 281 patients. Ann Surg Oncol 2013; 20:3618–25. 23838913 10.1245/s10434-013-3089-0

[66] Guo W, Chen M, Dusza S et al Predilection of acral lentiginous melanoma for sites of chronic mechanical stress: a meta-analytic review. J Am Acad Dermatol 2025; 93:AB6. 10.1016/j.jaad.2025.09.102 41076130

[67] Lallas A, Kyrgidis A, Koga H et al The BRAAFF checklist: a new dermoscopic algorithm for diagnosing acral melanoma. Br J Dermatol 2015; 173:1041–9. 26211689 10.1111/bjd.14045

[68] Braun RP, Thomas L, Dusza SW et al Dermoscopy of acral melanoma: a multicenter study on behalf of the international dermoscopy society. Dermatology 2014; 227:373–80. 10.1159/000356178 24296632

[69] Fernandez-Flores A, Cassarino DS. Histopathological diagnosis of acral lentiginous melanoma in early stages. Ann Diagn Pathol 2017; 26:64–9. 27601330 10.1016/j.anndiagpath.2016.08.005

[70] Phan A, Touzet S, Dalle S et al Acral lentiginous melanoma: a clinicoprognostic study of 126 cases. Br J Dermatol 2006; 155:561–9. 16911282 10.1111/j.1365-2133.2006.07368.x

[71] Wang M, Fukushima S, Sheen YS et al The genetic evolution of acral melanoma. Nat Commun 2024; 15:6146. 39034322 10.1038/s41467-024-50233-z PMC11271482

[72] Yeh I, Jorgenson E, Shen L et al Targeted genomic profiling of acral melanoma. J Natl Cancer Inst 2019; 111:1068–77. 30657954 10.1093/jnci/djz005 PMC6792090

[73] Namikawa K, Yamazaki N. Targeted therapy and immunotherapy for melanoma in Japan. Curr Treat Options Oncol 2019; 20:7. 30675668 10.1007/s11864-019-0607-8 PMC6344396

[74] Nakamura Y, Fujisawa Y. Diagnosis and management of acral lentiginous melanoma. Curr Treat Options Oncol 2018; 19:42. 29951919 10.1007/s11864-018-0560-y

[75] Dugan MM, Perez MC, Karapetyan L et al Management of acral lentiginous melanoma: current updates and future directions. Front Oncol 2024; 14:1323933. 38390259 10.3389/fonc.2024.1323933 PMC10882087

[76] Jung S, Johnson DB. Management of acral and mucosal melanoma: medical oncology perspective. Oncologist 2022; 27:703–10. 35640549 10.1093/oncolo/oyac091 PMC9355814

[77] Basurto-Lozada P, Molina-Aguilar C, Castaneda-Garcia C et al Acral lentiginous melanoma: basic facts, biological characteristics and research perspectives of an understudied disease. Pigment Cell Melanoma Res 2021; 34:59–71. 32330367 10.1111/pcmr.12885 PMC7818404

[78] Bradford PT, Goldstein AM, McMaster ML et al Acral lentiginous melanoma: incidence and survival patterns in the United States, 1986-2005. Arch Dermatol 2009; 145:427–34. 19380664 10.1001/archdermatol.2008.609 PMC2735055

[79] Saida T, Miyazaki A, Oguchi S et al Significance of dermoscopic patterns in detecting malignant melanoma on acral volar skin: results of a multicenter study in Japan. Arch Dermatol 2004; 140:1233–8. 15492186 10.1001/archderm.140.10.1233

[80] Fracaroli TS, Lavorato FG, Maceira JP et al Parallel ridge pattern on dermoscopy: observation in non-melanoma cases. An Bras Dermatol 2013; 88:646–8. 24068145 10.1590/abd1806-4841.20132058 PMC3760949

[81] Criscito MC, Stein JA. Improving the diagnosis and treatment of acral melanocytic lesions. Melanoma Manag 2017; 4:113–23. 30190914 10.2217/mmt-2016-0017 PMC6094663

[82] Steere AC, Strle F, Wormser GP et al Lyme borreliosis. Nat Rev Dis Primers 2016; 2:1–9. 10.1038/nrdp.2016.90 PMC5539539 27976670

[83] Stanek G, Wormser GP, Gray J et al Lyme borreliosis. Lancet 2012; 379:461–73. 21903253 10.1016/S0140-6736(11)60103-7

[84] Starace M, Alessandrini A, Piraccini BM et al Adherent serous crust of the scalp: inflammatory or infectious hair disease? A case of scalp eschar and neck lymph adenopathy after a tick bite. Biomed J Sci Tech Res 2021; 35:27596–8.

[85] Dika E, Patrizi A, Veronesi G et al Malignant cutaneous tumours of the scalp: always remember to examine the head. J Eur Acad Dermatol Venereol 2020; 34:2208–15. 32119157 10.1111/jdv.16330

[86] Pereira AR, Collgros H, Guitera P et al Melanomas of the scalp: is hair coverage preventing early diagnosis? Int J Dermatol 2021; 60:340–6. 33128467 10.1111/ijd.15283

[87] Cardenas-de la Garza JA, De la Cruz-Valadez E, Ocampo-Candiani J et al Clinical spectrum of Lyme disease. Eur J Clin Microbiol Infect Dis 2019; 38:201–8. 30456435 10.1007/s10096-018-3417-1

[88] Stupica D, Maraspin V, Bogovič P et al Comparison of clinical course and treatment outcome for patients with early disseminated or early localized Lyme borreliosis. JAMA Dermatol 2018; 154:1050–6. 30073319 10.1001/jamadermatol.2018.2306 PMC6143036

[89] Lantos PM, Rumbaugh J, Bockenstedt LK et al Clinical practice guidelines by the Infectious Diseases Society of America (IDSA), American Academy of Neurology (AAN), and American College of Rheumatology (ACR): 2020 guidelines for the prevention, diagnosis and treatment of Lyme disease. Clin Infect Dis 2021; 72:e1–48. 33417672 10.1093/cid/ciaa1215

[90] Shapiro ED, Wormser GP. Lyme disease in 2018: what is new (and what is not). JAMA 2018; 320:635–6. 30073279 10.1001/jama.2018.10974

[91] Kobayashi T, Auwaerter PG. Diagnostic testing for Lyme disease. Infect Dis Clin North Am 2022; 36:605–20. 36116838 10.1016/j.idc.2022.04.001

[92] Kenyon SM, Chan SL. A focused review on Lyme disease diagnostic testing: an update on serology algorithms, current ordering practices, and practical considerations for laboratory implementation of a new testing algorithm. Clin Biochem 2023; 117:4–9. 34875253 10.1016/j.clinbiochem.2021.12.001

[93] Nguyen CT, Cifu AS, Pitrak D. Prevention and treatment of Lyme disease. JAMA 2022; 327:772–3. 35191942 10.1001/jama.2021.25302

[94] Cameron D, Gaito A, Harris N et al Evidence-based guidelines for the management of Lyme disease. Expert Rev Anti Infect Ther 2004; 2:S1–13. 15581390 10.1586/14789072.2.1.s1

[95] Seltzer EG, Gerber MA, Cartter ML et al Long-term outcomes of persons with Lyme disease. JAMA 2000; 283:609–16. 10665700 10.1001/jama.283.5.609

[96] Chung JE . Medical dramas and viewer perception of health: testing cultivation effects. Hum Commun Res 2014; 40:333–49.

[97] Sonego A, Rocchi M. Medical drama TV series: a semi-systematic literature review. Online J Commun Media Technol 2024; 14:1–29.

[98] Rigopoulos D, Larios G, Katsambas A. Skin signs of systemic diseases. Clin Dermatol 2011; 29:531–40. 21855729 10.1016/j.clindermatol.2010.09.021

[99] Hoffman BL, Shensa A, Wessel C et al Exposure to fictional medical television and health: a systematic review. Health Educ Res 2017; 32:107–23. 28334962 10.1093/her/cyx034

[100] Tan NN, Soeselo DA, Puspadewi N et al From screen to stethoscope: how medical dramas impact the motivation of aspiring medical students. Asia Pac Sch 2025; 10:58–64.

[101] Terry D, Peck B. Television as a career motivator and education tool: a final-year nursing student cohort study. Eur J Investig Health Psychol Educ 2019; 10:346–57. 10.3390/ejihpe10010026 PMC8314239 34542489

[102] Hoffman BL, Hoffman R, Wessel CB et al Use of fictional medical television in health sciences education: a systematic review. Adv Health Sci Educ Theory Pract 2018; 23:201–16. 28083814 10.1007/s10459-017-9754-5

